# *Staphylococcus aureus* β-Hemolysin Up-Regulates the Expression of IFN-γ by Human CD56^bright^ NK Cells

**DOI:** 10.3389/fcimb.2021.658141

**Published:** 2021-03-29

**Authors:** Zhangchun Guan, Yu Liu, Chenghua Liu, Huiting Wang, Jiannan Feng, Guang Yang

**Affiliations:** ^1^ Beijing Institute of Pharmacology and Toxicology, Beijing, China; ^2^ State Key Laboratory of Toxicology and Medical Countermeasures, Beijing, China

**Keywords:** *S. aureus*, β-hemolysin, IFN-γ, CD56^bright^ NK cells, ERK, calcium

## Abstract

IFN-γ is produced upon stimulation with *S. aureus* and may play a detrimental role during infection. However, whether hemolysins play a role in the mechanism of IFN-γ production has not been fully characterized. In this study, we demonstrated that Hlb, one of the major hemolysins of *S. aureus*, upregulated IFN-γ production by CD56^bright^ NK cells from human peripheral blood mononuclear cells (PBMCs). Further investigation showed that Hlb increased calcium influx and induced phosphorylation of ERK1/2. Either blocking calcium or specifically inhibiting phosphorylation of ERK1/2 decreased the production of IFN-γ induced by Hlb. Moreover, we found that this process was dependent on the sphingomyelinase activity of Hlb. Our findings revealed a novel mechanism of IFN-γ production in NK cells induced by Hlb, which may be involved in the pathogenesis of *S. aureus*.

## Introduction

*Staphylococcus aureus* (*S. aureus*) is an important pathogen that causes a wide range of infections, including skin infections, endocarditis or life-threatening septicemia, and pneumonia (Tong et al., 2015; Oliveira et al., 2018). *S. aureus* produces four hemolysins: α-, β-, γ-, and δ-hemolysin, which are involved in the development of infectious diseases (Dinges et al., 2000; Bownik and Siwicki, 2008). α-, γ-, and δ-hemolysin are pore-forming toxins (PFT) that can induce target cell lysis by forming pores in the cell membrane (Talbot et al., 2001; Wilke and Bubeck Wardenburg, 2010; Powers et al., 2012; Spaan et al., 2014; Spaan et al., 2015; von Hoven et al., 2019). However, β-hemolysin (Hlb) is a non-pore-forming toxin (Vandenesch et al., 2012). As a neutral sphingomyelinase (SMase), Hlb can specifically degrade sphingomyelin into ceramide and choline phosphate, which is essential for target cell membrane damage (Huseby et al., 2007; Vandenesch et al., 2012). It has also been reported that Hlb forms covalent cross-links in the presence of DNA, which strongly stimulates biofilm formation in a rabbit model of infectious endocarditis (Huseby et al., 2007; Huseby et al., 2010; Katayama et al., 2013).

IFN-γ is an important cytokine produced by several cells involved in innate and adaptive immunity, including natural killer (NK) cells, natural killer T cells, cytotoxic T cells, and Th1 cells (Mojic et al., 2017; Burke and Young, 2019). During *S. aureus* infection, IFN-γ is produced and cooperatively regulates host resistance with other endogenous inflammatory cytokines (Nakane et al., 1995; Culshaw et al., 2005). However, it has been observed that IFN-γ-deficient mice have higher survival rates than wild-type mice during *S. aureus* infection (Sasaki et al., 2000). Neutralizing IFN-γ with anti-IFN-γ monoclonal antibodies also increased the survival rates of infected mice (Nakane et al., 1995). These results suggest that the production of IFN-γ induced by *S. aureus* is detrimental in murine models.

Yoshihara et al. reported that *S. aureus* stimulated the production of IFN-γ by NK cells in human PBMCs (Yoshihara et al., 1993). Moreover, the superantigen of staphylococcal enterotoxin A (SEA) expressed by *S. aureus* increased the expression of IFN-γ by FOXP3+ T helper cells, which was partially mediated in a monocyte-dependent manner (Björkander et al., 2016). In this study, we explored whether *S. aureus* hemolysins are involved in the production of IFN-γ. Hlb was found to induce the production of IFN-γ by human CD56^bright^ NK cells. The increase in IFN-γ production was mediated by increased calcium and ERK1/2 phosphorylation. Furthermore, the SMase activity of Hlb was found to be essential for the production of IFN-γ.

## Materials and Methods

### Cells and Cell Culture

The NK-92 cell line was originally established from the PBMCs of a 50-year-old male patient with rapidly progressive non-Hodgkin’s lymphoma (Gong et al., 1994). As a CD56^bright^ NK cell line, NK-92 cells retain some crucial functional features of CD56^bright^ NK cells, including KIRs expression (Trundley et al., 2006; Wu et al., 2020), granzyme K expression (Jiang et al., 2011), and cytokine expression (Wang et al., 2018). In this study, NK-92 cells were purchased from the China Infrastructure of Cell Line Resources. Cells were grown in α-MEM (Gibco) supplemented with 12.5% (v/v) FBS, 12.5% (v/v) horse serum, 55 mM β-mercaptoethanol, 500 U/mL IL-2, 100 U/mL penicillin, and streptomycin at 37°C in 5% CO_2_.

Following informed consent, PBMCs were freshly isolated from venous blood of healthy donors by density gradient centrifugation as described previously (Higdon et al., 2016). Fresh human whole blood was collected in tubes containing anticoagulants. Lymphocyte separation medium (TBD) was added to a fresh 15 mL conical tube, and the same volume of fresh blood was added carefully to avoid mixing the two layers. Next, the tube was centrifuged at 800 g for 30 min at room temperature. The second layer of the buffy coat was the mononuclear cell band. PBMCs were washed twice with PBS before use. Cells were grown in RPMI-1640 (Gibco) supplemented with 10% (v/v) FBS, 100 U/mL penicillin, and streptomycin at 37°C in 5% CO_2_.

Primary CD56^bright^ NK cells were isolated from human PBMCs. The PBMCs were stained with antibodies: PE anti-CD56 (clone: MEM-188) and APC anti-CD3 (clone: HIT3a) (BioLegend) for 30 min at room temperature in the dark. CD56^bright^ CD3- cells were collected using flow cytometry (BD Aria II).

### Purification of Recombinant Hlb and the Site-Specific Mutants

The *hlb* gene (gene ID: 3238648) was amplified from the genome of *S. aureus* COL using the following primers:

Hlb(F): GCCATATGGCTAGCGAATCTAAGAAAGATGATAC,
Hlb(R): CACCACCCACTCGAGCTATTTACTATAGGCTTTGA,
Hlb_H161A_(F1): GCCATATGGCTAGCGAATCTAAGAAAGATGATAC,
Hlb_H161A_(R1): CGTTGTGGTGCTGGA**GC**TGATCGAAAAATTAG,
Hlb_H161A_(F2): CTAATTTTTCGATCA**GC**TCCAGCACCACAACG,
Hlb_H161A_(R2): CACCACCCACTCGAGCTATTTACTATAGGCTTTGA,
Hlb_H149N_(F1): GCCATATGGCTAGCGAATCTAAGAAAGATGATAC,
Hlb_H149N_(R1): CTTCAGATTGTGTAT**T**TGTACCGATAACGTGAACG,
Hlb_H149N_(F2): CGTTCACGTTATCGGTACA**A**ATACACAATCTGAAG,
Hlb_H149N_(R2): CACCACCCACTCGAGCTATTTACTATAGGCTTTGA,
Hlb_H288N_(F): GCCATATGGCTAGCGAATCTAAGAAAGATGATAC,
Hlb_H288N_(R):GCGCCGCTCGAGCTATTTACTATAGGCTTTGATTGGGTAAT**T**ATCTGAAAAATATT.

The underlined sequences GCTAGC and CTCGAG indicate the *Nhe1* and *Xho1* recognition sites, respectively. The bold letters indicate the mutation sites. The complete Hlb_H161A_ and Hlb_H149N_ genes were obtained by overlap PCR. The amplified fragments were digested with *Nhe1* and *Xho1* and inserted into the expression vector pET-28(a). Recombinant proteins were expressed in *Escherichia coli* (BL21) and purified using Ni Sepharose™ 6 Fast Flow (GE Healthcare). Purified proteins were passed through a Pierce High-Capacity Endotoxin Removal Resin (Thermo) to remove endotoxins.

### Affinity Purification of Anti-Hlb Antibody

Polyclonal antibodies against Hlb were generated from New Zealand white rabbits. Rabbits were immunized with Hlb once a month, three times. The first injection was performed using 500 μL Hlb (1 mg/mL) with the same volume of Freund’s complete adjuvant (Sigma). Freund’s complete adjuvant was replaced with Freund’s incomplete adjuvant (Sigma) for the following two booster injections. After two weeks, serum was collected and rabbit antibodies against Hlb were purified using the Hlb conjugated resin as following. Recombinant Hlb was coupled with CNBr Sepharose 4B (GE Healthcare) according to the manufacturer’s instructions. The serum was then incubated with the coupling sepharose for 1 h at room temperature. Next, the coupling sepharose was washed with PBS six times. The antibody was eluted with 0.2 M Gly-HCl (pH2.4), and then neutralized with 1 M Tris-HCl (pH9.1).

All animal experimental protocols of the study are in accordance with the national guidelines for the use of animals in scientific research “Regulations for the Administration of Affairs Concerning Experimental Animals” and were approved by the Animal Care and Use Committee of Beijing Institute of Pharmacology and Toxicology.

### Measurement of IFN-γ Production

For the ELISA assay, cells were incubated with a given concentration of proteins for the indicated hours at 37°C. IFN-γ in the supernatant was determined using a human IFN-γ ELISA assay kit (BioLegend) according to the manufacturer’s instructions.

For the flow cytometry assay, human PBMCs were incubated with Hlb for 12 h at 37°C. Brefeldin A (BFA) was added and the cells were continually cultured for 6 h. The treated cells were harvested and stained with antibodies: PE anti-CD56 (clone: MEM-188) and APC anti-CD3 (clone: HIT3a) (BioLegend) for 30 min at room temperature in the dark. Next, cells were washed and fixed with Fixation/Permeabilization working solution (eBioscience) for 30 min at 4°C in the dark. Cells were then washed with permeabilization buffer and stained with PerCP-Cy5.5 anti-IFN-γ antibody (clone: 4S.B3) or PerCP-Cy5.5 mouse IgG1 κ isotype control antibody (clone: MOPC-21) (BioLegend) for 30 min at room temperature in the dark. Finally, the cells were washed with permeabilization buffer and resuspended in PBS. Analysis was performed using FlowJo software (TreeStar).

For quantitative reverse transcription PCR (qRT-PCR) assays, total RNA was isolated from NK-92 cells that had already been incubated with 2 μg/mL Hlb for 12 h at 37°C using the TransZol Up Plus RNA Kit (TransGen). Next, complementary DNA was synthesized using a Revertra Ace qPCR Kit (Toyobo) and analyzed using the Bio-Rad iQ5 Real Time PCR System.

### Hemolytic Activity Assays

Fresh sheep red blood cells were washed three times and diluted 50 times with PBS. Hlb was diluted to different final concentrations with 1% BSA-PBS. Then, sheep red blood cells were incubated with an equal volume of Hlb at 37°C for 1 h, followed by 4°C for 1 h. The released hemoglobin in the supernatant was collected by centrifugation and determined at 405 nm.

### Western Blotting

Cells were treated in α-MEM containing 2 μg/mL Hlb for the indicated minutes. To extract total cell protein, cells were harvested after washing with cold PBS and lysed with cold RIPA lysis buffer containing protease and phosphatase inhibitors. The samples were resolved on a 12% SDS-PAGE and electroblotted onto polyvinylidene fluoride (PVDF) membranes. Anti-STAT5A Ab, phospho-STAT5A Ab (BBI Life Sciences), phospho-STAT4 Ab, STAT4 rabbit mAb, NF-κB p65 rabbit mAb, phospho-NF-κB p65 rabbit mAb, p44/42 MAPK rabbit mAb, phospho-p44/42 MAPK rabbit mAb (Cell Signaling Technology), anti-GAPDH, and horseradish peroxidase-conjugated goat antibodies against rabbit IgG and mouse IgG (Jackson ImmunoResearch Laboratories) were used. Finally, the blots were detected using Pierce™ ECL Western Blotting Substrate (Thermo).

### Measurement of Intracellular Calcium

NK-92 cells were washed three times with HBSS (Beyotime) and incubated with 5 μM Fluo-3-AM (Beyotime) at 37°C for 45 min. Next, the cells were washed with HBSS three times and resuspended in 500 μL HBSS (containing Ca^2+^, Mg^2+^) supplemented with 10 mM HEPES (pH 7.5) and 1 mg/mL BSA. Finally, cells were analyzed using flow cytometry (BD Aria II) for 5 min.

### Detection of NK-92 Cell Binding With Hlb

NK-92 cells were incubated with 10 μg/mL Hlb in α-MEM (Gibco) for 1 h at 37°C. The cells were then collected and washed with 1% BSA-PBS by centrifugation. Next, the cells were incubated with an anti-6xHis tag antibody (FITC) in 1% BSA-PBS for 30 min at room temperature. Finally, the cells were washed with PBS and analyzed using flow cytometry (BD Aria II).

### Measurement of Lymphocyte Death

Human PBMCs were incubated with Hlb, HlgA/B, or Hla at 37°C for 12 h. The cells were then collected and stained with 7-AAD (BioLegend) according to the manufacturer’s instructions. The treated cells were analyzed using flow cytometry (BD Aria II) within 30 min.

### Construction of *hlb*-Deletion Mutant (COL_Δhlb_) From the *S. aureus* COL

The mutant was constructed based on homologous recombination (Brückner, 1997). The gene fragments upstream and downstream of the *hlb* gene from *S. aureus* COL genomic DNA were amplified using PCR. The kanamycin resistance gene from *S. aureus* 8325-4_Δ_ *_Vrax_* was also amplified using PCR. The pBT2 plasmid containing a homologous recombination fragment (upstream gene—kanamycin resistance gene—downstream gene) was constructed using enzyme digestion and ligation. Next, the plasmid was electroporated into COL. Homologous recombination between the bacterial genome and plasmid was induced by changing the temperature and kanamycin concentration. The mutant strains were screened by growing on plates containing kanamycin and chloramphenicol. Strains that grew on the kanamycin plate but not on the kanamycin and chloramphenicol plates were verified using PCR.

*S. aureu*s COL and COL_Δ_ *_hlb_* were individually cultured in BHI medium (BD) at 37°C for 12 h. The supernatant was collected and dialyzed in distilled water. It was then lyophilized and re-dissolved with 1/10 volume of PBS and concentrated 10 times.

### Statistical Analysis

GraphPad Prism software was used for data presentation and statistical analyses. Statistical analyses were performed using one-way analysis of variance (ANOVA). All experiments were repeated at least three times independently, and the data are expressed as the mean ± standard deviation (s.d). Statistical significance was set at P < 0.05.

## Results

### Hlb Enhanced the Production of IFN-γ in CD56^bright^ NK Cells in Human Peripheral Blood Mononuclear Cells

**Figure 1:**
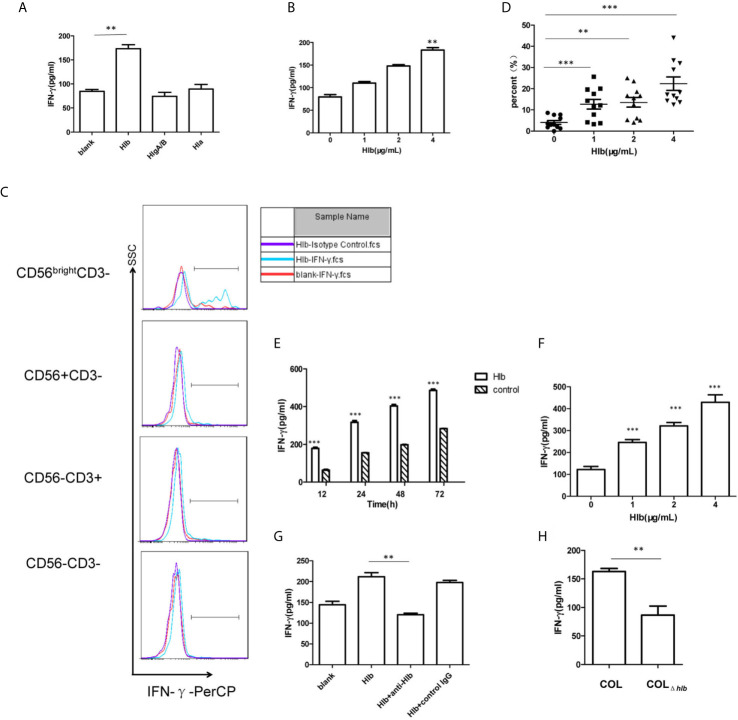
Hlb enhanced the production of IFN-γ in CD56^bright^ NK cells. **(A, B)** Detection of IFN-γ in the culture supernatant of human PBMC. Human P BMC were collected and incubated with different hemolysins (4 µ g/ml Hlb, 4 µ g/ml HlgA/B, and 0.25 µ g/ml Hla) **(A)** or with different concentrations of Hlb protein **(B)** respectively. The level of IFN-γ production in cell supernatant was determined by ELISA. **(C)** Detection of the expression of intracellular IFN-γ in Hu man PBMC. Human PBMC were incubated with Hlb (1 µ g/ml) for 18h and stained with anti-CD3, anti-CD56 and anti-IFN-γ (or Isotype Control), then the expression of intracellular IFN-γ in different cell populations was analyzed by flow cytometry. **(D)** PBMC from 11 healthy donors were collected individually and incubated with different concentrations of Hl b, respectively. Then the cells stained with anti-CD3, anti-CD56 and anti-IFN-γ. The percentage of IFN-γ + cells in CD56bright NK cells was analyzed (n=11). **(E–G)** Detection of IFN-γ production by NK-92 cells. NK-92 cells were incubated with different concentrations of Hlb for 12h **(E)** or incubated with Hlb (2 µ g/ml) for different time **(F)** Hl b was incubated with anti-Hlb (or control IgG) for 30min, then the mixture were added to culture medium of NK-92 cells for 12h **(G)**. The level of IFN-γ product ion in the cell supernatant was detected by ELISA. **(H)** Detection of IFN-γ production by NK-92 cells induced by the bacterial supernatant. NK-92 cells were incubated with 5 µ l concentrated supernatant (10X) of S. aureus COL or hlb-deletion COL (COL∆hlb) for 12h, respectively. The level of IFN-γ production in the cell supernatant was determined by ELISA. ** means P<0.01, *** means P<0.001.

As *S. aureus* stimulated the production of IFN-γ in human PBMCs (Yoshihara et al., 1993; Björkander et al., 2016) and hemolysins-assisted immune escape during the pathogenesis of *S. aureus* (Bownik and Siwicki, 2008; Wilke and Bubeck Wardenburg, 2010; von Hoven et al., 2019), we determined that *S. aureus* hemolysins are involved in IFN-γ production. We expressed and purified three kinds of hemolysins, Hla, Hlb, and HlgA/HlgB (**Supplementary Material S1**). The purified toxins were then incubated with human PBMCs. The results of flow cytometry indicated that these hemolysins at the given certain concentration (4 μg/mL Hlb, 4 μg/mL HlgA/B, or 0.25 μg/mL Hla) did not induce lymphocyte death (**Supplementary Material S2**). The production of IFN-γ by PBMCs after incubation with different toxins was determined using ELISA. Hlb stimulated the production of IFN-γ, while the other two hemolysins had no effect (**Figure 1A**). Moreover, the production of IFN-γ stimulated by Hlb was concentration-dependent (**Figure 1B**). IFN-γ production in the different cell subsets of PBMCs was further detected by staining for CD56 and CD3 with specific antibodies. It was shown that the upregulated IFN-γ expression was observed in CD56^bright^ NK cells [CD56^bright^ CD3^-^] after human PBMCs were incubated with Hlb (**Figure 1C**), and Hlb was found to increase the percentage of IFN-γ-producing cells in CD56^bright^ NK cells in a concentration-dependent manner (**Figure 1D**). Human NK cells can be subdivided into two subsets (CD56^bright^ and CD56^dim^ NK cells) based on the relative expression of CD56. CD56^bright^ NK cells are considered efficient cytokine producers endowed with immunoregulatory properties, but they can also become cytotoxic upon appropriate activation (Poli et al., 2009; Michel et al., 2016; Wagner et al., 2017).

NK-92 is a CD56^bright^ NK cell line that retains some crucial functional features of CD56^bright^ NK cells, including cytokine expression (Gong et al., 1994; Wang et al., 2018). Therefore, we used NK-92 cells for further mechanistic investigations. As expected, Hlb induced a marked increase in IFN-γ production by NK-92 cells in a concentration-and time-dependent manner (**Figures 1E, F**). Moreover, we found that the level of IFN-γ mRNA in NK-92 cells increased after incubation with Hlb (**Supplementary Material S3**). Rabbit polyclonal antibodies against Hlb (anti-Hlb) were prepared (**Supplementary Material S4**). Further investigation demonstrated that anti-Hlb blocked IFN-γ production induced by Hlb (**Figure 1G**).

*S. aureus* COL is a methicillin-resistant *Staphylococcus aureus* (MRSA) strain with a known complete genome. It was reportedly isolated in the early 1960s from the operating theater in a hospital in Colindale, England (Gill et al., 2005). To further confirm whether Hlb induced the production of IFN-γ in NK-92 cells, an *hlb*-deletion mutant (COL*_Δhlb_*) from *S. aureus* COL was generated. The supernatant collected from COL or COL*_Δhlb_* was incubated with NK-92 cells. The level of IFN-γ stimulated by COL supernatant was significantly higher than that stimulated by COL*_Δhlb_* supernatant (**Figure 1H**).

### Phosphorylation of ERK1/2 Modulated IFN-γ Production Induced by Hlb

**Figure 2:**
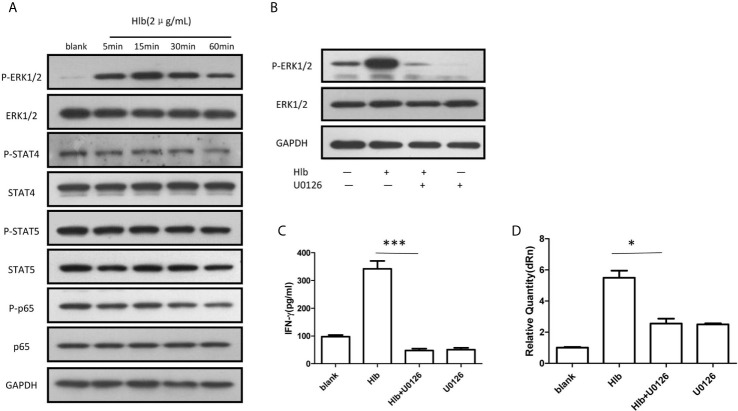
Phosphorylation of ERK1/2 modulated IFN-γ production induced by Hlb. **(A)** Phosphorylation of different signal molecules was detected by western blotting. NK-92 cell s were incubated with Hlb (2 µ g/ml) for 5, 15, 30 and 60mins, respectively. The cell s were lysed and anti-ERK l/2, anti-P-ERK l /2, anti-STAT4, anti-P-STAT4, anti­STAT5, anti-P-STAT5 and an ti-NF-κB (p65), anti-P-NF-κB (p65) were used to identify the corresponding proteins. **(B)** NK-92 cells were incubated with Hlb in the presence of U0126 (10 µ M) for 5mins, the phosphorylation of ERK l/2 were detected by western blotting. **(C, D)** Detection of IFN-γ production by NK-92 cells. NK-92 cells were incubated with Hlb in the presence of U0126 (10 µ M) for 12h , then the IFN-γ in supernatant was determined by ELISA **(C)** and IFN-γ mRNA expression was detected by qRT-PCR **(D)**. * means P<0.05, *** means P<0.001.

It has been reported that the activation of several intracellular molecules, including STAT4 (Matikainen et al., 2001; Hagberg et al., 2018), STAT5 (Gotthardt et al., 2016), NF-κB (Vulpis et al., 2017), and ERK1/2 (Yu et al., 2000) regulate the transcription of IFN-γ in NK cells. Therefore, the phosphorylation of these molecules was measured in NK-92 cells. It was found that phosphorylation of ERK1/2 but not STAT4, STAT5, or NF-κB (p65) was increased (**Figure 2A**), and the phosphorylation of ERK1/2 stimulated by Hlb was blocked by the MEK1/2 inhibitor U0126 (**Figure 2B**). In line with our expectations, the increased level of IFN-γ mRNA and the elevated IFN-γ production in NK-92 cells induced by Hlb was blocked by U0126 (**Figures 2C, D**).

### Intracellular Calcium Was Increased by Hlb

**Figure 3:**
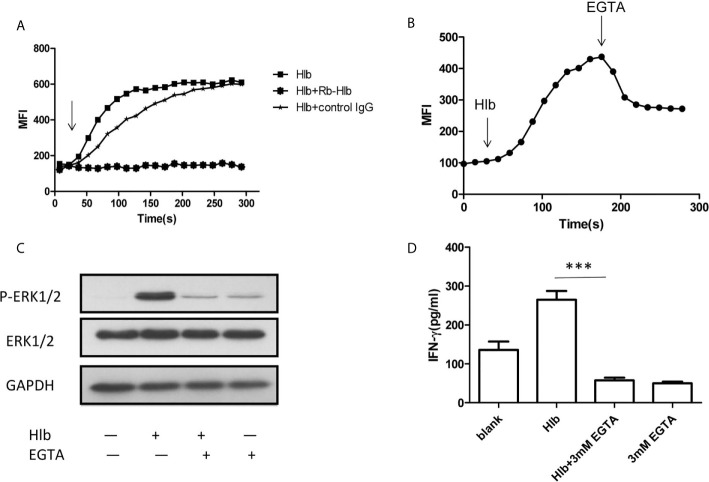
Intracellular calcium was increased by Hlb. **(A, B)** Detection of intracellular calcium in NK-92 cells. NK-92 cells were incubated with 5 µ M Fluo-3-AM for 45mins. The fluorescent signal of NK-92 cells were detected for 20s, then Hlb or the mixture (Hlb and anti­Hlb/control IgG) was added immediately, the fluorescent signal was detected continuously to 5 minutes by flow cytometry **(A)** EGTA was added immediately at 160s after addition of Hlb, the fluorescent signal was collected continuously to 5 minutes **(B)**. MFI represents the average intracellular fluorescence intensity, reflecting the amount of intracellular calcium. **(C)** Phosphorylation of ERK was detected by western blotting. NK-92 cells were incubated with Hlb (2 µ g/ml) in the presence of EGTA (3mM) for 5mins, the cells were lysed and anti-ERK1/2 and anti-P-ERK1/2 were used to identify the corresponding proteins. **(D)** Detection of IFN-γ production by NK-92 cells. NK-92 cells were incubated with Hlb (2 µ g/ml) in the presence of EGTA (3mM) for 12h. The IFN-γ in supernatant was determined by ELISA. *** means P<0.001.

As α-hemolysin and γ-hemolysin are reported to bind specific receptors on cell membranes (Wilke and Bubeck Wardenburg, 2010; Spaan et al., 2014), we wondered whether Hlb also directly bond to CD56^bright^ NK cells. The results of flow cytometry showed that Hlb did not bind to NK-92 cells (**Supplementary Material S5**). Given that intracellular calcium induces the activation of ERK1/2 (Agell et al., 2002; Gong et al., 2019), we explored whether Hlb increased intracellular calcium in NK-92 cells. It was found that the level of intracellular calcium immediately increased with the addition of Hlb (**Figure 3A**). However, the intracellular calcium concentration was not elevated when Hlb was pre-incubated with anti-Hlb (**Figure 3A**). Ethylene glycol-bis(2-aminoethylether)-*N,N’,N’,N’*-tetraacetic acid (EGTA) is a specific chelator of Ca^2+^. The intracellular calcium stimulated by Hlb was partially blocked when EGTA was added (**Figure 3B**). In addition, ERK1/2 phosphorylation and IFN-γ production induced by Hlb were blocked by EGTA addition (**Figures 3C, 3D**).

### IFN-γ Expression Induced by Hlb Is Dependent on Its SMase Activity

**Figure 4:**
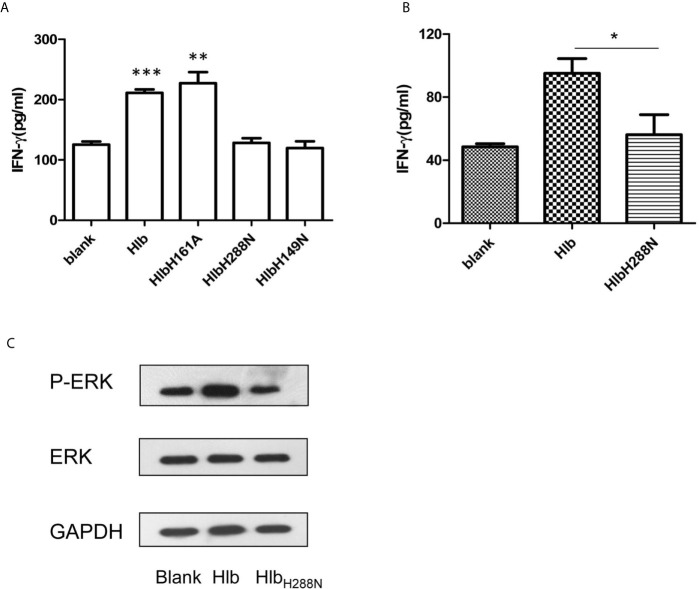
Up-regulating IFN-γ expression by Hlb is dependent on its SMase activity. **(A, B)** Detection of IFN-γ production. NK-92 cells were incubated with Hlb, HlbH288N, HlbH149N or HlbH161A (2 µ g/ml) for 12h, respectively **(A)** Primary CD56bright NK cells were incubated with Hlb or HlbH288N at 37ºC for 12 hours **(B)**. The IFN-γ in supernatant was determined by ELISA. **(C)** Primary CD56bright NK cells were incubated with Hlb or HlbH288N at 37ºC for 10 minutes. The phosphorylation of ERK1/2 was detected by western blotting. * means P<0.05, ** means P<0.01, *** means P<0.001.

Hlb is a neutral SMase and a DNA biofilm ligase. To further explore the mechanisms of action that contributed to the increased production of IFN-γ induced by Hlb, we constructed and purified recombinant SMase-deficient Hlb mutants (Hlb_H288N_, Hlb_H149N_) and biofilm ligase-deficient Hlb mutants (Hlb_H161A_) (Huseby et al., 2007; Herrera et al., 2016; Herrera et al., 2017) (**Supplementary Material S6**). As a neutral SMase, Hlb cleaves sphingomyelin and induces lysis of sheep red blood cells (RBCs) (Doery et al., 1965; Vandenesch et al., 2012). We analyzed the released hemoglobin in the supernatant of sheep RBCs after incubation with these mutants. Hlb and Hlb_H161A_ were found to lyse sheep RBCs at a low concentration (8 ng/mL). However, sheep RBCs were resistant to lysis mediated by either Hlb_H288N_ or Hlb_H149N_ (**Supplementary Material S7**). IFN-γ production was analyzed in NK-92 cells after incubation with these mutants. The results showed that Hlb_H288N_ and Hlb_H149N_ failed to induce IFN-γ production in NK-92 cells, while Hlb_H161A_ had an effect similar to that of Hlb (**Figure 4A**).

U0126 and EGTA may directly inhibit the SMase activity of Hlb, thereby blocking the production of IFN-γ induced by Hlb. Therefore, we investigated whether U0126 and EGTA inhibited the SMase activity of Hlb. Sheep RBCs were incubated with Hlb in the presence of U0126 or EGTA, and the released hemoglobin in the supernatant was measured. The results showed that the lysis of sheep RBCs mediated by Hlb was not altered by the addition of U0126 or EGTA (**Supplementary Material S8a, S8b**).

For further investigation, the primary CD56^bright^ NK cells in human PBMCs from healthy donors were isolated and incubated with Hlb or Hlb_H288N_. Consistent with the observation in NK-92 cells, the production of IFN-γ by primary CD56^bright^ NK cells was enhanced by incubation with Hlb but not Hlb_H288N_ (**Figure 4B**). Meanwhile, it was observed that the phosphorylation of ERK in CD56^bright^ NK cells was induced by Hlb (**Figure 4C**).

## Discussion

As a hemolysin expressed by *S. aureus*, Hlb, a non-PFT hemolysin, is a neutral SMase. Here, we found that Hlb induced the production of IFN-γ in human CD56^bright^ NK cells. Previous studies have shown that the combination of IL-12 and IL-18 is usually used to induce the production of IFN-γ in CD56^bright^ NK cells, which is mediated by the activation of STAT4 (Thierfelder et al., 1996; Fehniger et al., 1999; Matikainen et al., 2001; Nguyen et al., 2002). However, we found that phosphorylation of ERK1/2, but not STAT4, was stimulated by Hlb, suggesting that the mechanism of IFN-γ production triggered by Hlb was different from that of other triggering factors.

IFN-γ is an important cytokine produced by several cells involved in innate and adaptive immunity, including natural killer (NK) cells, natural killer T cells, cytotoxic T cells, and Th1 cells. However, the production of IFN-γ in these cells is regulated by different mechanisms. In our study, we observed that the production of IFN-γ induced by Hlb was only in CD56^bright^ NK cells. We speculated that the production of IFN-γ in CD56^bright^ NK cells was more susceptible to the degradation of sphingomyelin induced by Hlb compared with other IFN-γ producing cells, which needs to be investigated in the future.

The sequenced genome revealed that *S.aureus* COL express enterotoxins (SEA, SEB, SEl-K and SEl-I), but did not express toxic shock syndrome toxin (Gill et al., 2005). It has been reported that enterotoxins characterized as the superantigens bind MHC-II molecules and coordinate to T cell receptors. *S.aureus* enterotoxins induce massive nonspecific proliferation of T cells, which leads to release of IFN-γ (Cameron et al., 2001). The direct effect of enterotoxins on NK cells has not been reported.

We performed flow cytometry to detect whether Hlb directly bond to NK-92 cells. It was found that Hlb did not directly bind to NK-92 cells, suggesting that there was no specific receptor for Hlb on the cell membrane. However, both Hlb_H288N_ and Hlb_H149N_ failed to stimulate the production of IFN-γ, which indicated that the SMase activity of Hlb played a crucial role in this process. We speculated that there might be two mechanisms for Hlb to stimulate calcium influx and upregulate the expression of IFN-γ through its sphingomyelinase activity. On one hand, ceramide might trigger intracellular signaling pathways leading to calcium influx. Sphingomyelin is one of the major components of mammalian cell membranes. In the presence of Mg^2+^, Hlb cleaves sphingomyelin into ceramide and phosphorylcholine. Ceramide is an intracellular signaling molecule that shows activity in many biological processes (Castro et al., 2014). Studies have shown that bacterial SMase can activate the sphingomyelin pathway directly (Kim et al., 1991). Bacterial SMase increases intracellular Ca^2+^ concentration in primary cultured cerebral vascular smooth muscle cells (Zheng et al., 2000). On the other hand, Ca^2+^ influx is regulated by ion channels located on the cell membrane (Redmond et al., 2017). It has been reported that the degradation of sphingomyelin mediated by SMase induces the formation of ceramide-enriched domains in cell membrane (Montes et al., 2008). Meanwhile, Hlb has been identified to induce the shedding of cell membrane proteins (Park et al., 2004). We assumed that the altered cell membrane and the shedding of cell membrane proteins mediated by Hlb might trigger Ca^2+^ influx. The mechanism in detail will be investigated in the future. In addition, neutral SMase is reported to be expressed in several bacteria, such as *Bacillus cereus*, *Listeria ivanovii* (Kim et al., 1991; Clarke et al., 2006). All of these SMases hydrolyze sphingomyelin into ceramide and phosphocholine, so they may also induce the response of NK cells and upregulate the expression of IFN-γ.

It is well known that most of human *S. aureus* isolates do not express functional Hlb, which is due to the integration of bacteriophages into the structural gene *hlb* (Pantucek et al., 2004; van Wamel et al., 2006). Therefore, it has been assumed that Hlb does not significantly contribute to pathogenesis during *S. aureus* infection. However, evidences demonstrate that in some conditions, precise excision of the bacteriophage occurs, leading to the re-production of Hlb. Phage excision with Hlb expression has been reported in *S. aureus* isolates from patients with cystic fibrosis (CF) (Goerke et al., 2004). Strain 8325-4φ13 and *S. aureus* isolated from CF patients harboring φ13-like phages could be detected as Hlb-positive after treatment with either ciprofloxacin or trimethoprim (Goerke et al., 2006). In the mouse ear skin colonization model, the spontaneous precise excision of the bacteriophage significantly improved the colonization ability of the MW2 strain (Katayama et al., 2013). Moreover, it has been identified that strains carrying phage φSa3 exist as heterogeneous populations containing a subset producing Hlb. The frequency of colonies producing Hlb in stains carrying phage φSa3 is dependent on the growth condition *in vitro* and *in vivo*. Re-producing of Hlb aggravates pneumonia and infective endocarditis in rabbit models, which suggests that Hlb contributes significantly to tissue disease progression (Salgado-Pabón et al., 2014). These studies suggest that during chronic infections, Hlb production may be beneficial for *S. aureus* to resist antibiotics and the host immune system, thus further promoting disease progression. We found that Hlb induced the production of IFN-γ in human CD56^bright^ NK cells. Previous studies have shown that the production of endogenous IFN-γ aggravates *S. aureus* infection *in vivo* (Nakane et al., 1995; Sasaki et al., 2000; Culshaw et al., 2005; Satorres et al., 2009). Therefore, the production of IFN-γ in NK cells induced by Hlb may be a new mechanism that aggravates *S. aureus* infection. As there is no cell subpopulation in mice corresponding to human CD56^bright^ NK cells, we did not investigate the effect of IFN-γ production induced by Hlb on *S. aureus* pathogenesis in a murine model.

In addition, *S. aureus* is widely colonized in the human body that is correlated with the development of many persistent severe inflammatory diseases, such as atopic dermatitis (Weidinger et al., 2018; Vickery et al., 2019), systemic lupus erythematosus (Conti et al., 2016), diabetes (Dryden et al., 2015), and insulin resistance (Liu et al., 2018). IFN-γ is reported to be involved in the development of these diseases (Klunker et al., 2003; Antonelli et al., 2014; Di Bari, 2015). Therefore, we speculate that the expression of Hlb during *S. aureus* colonization may contribute to chronic inflammatory diseases. In summary, we have identified that β-hemolysin directly upregulates the expression of IFN-γ in human CD56^bright^ NK cells, which is dependent on its SMase activity. Our findings present an additional novel mechanism of IFN-γ production induced by *S. aureus*, which may be involved in its pathogenesis.

## Data Availability Statement

The original contributions presented in the study are included in the article/**Supplementary Material**. Further inquiries can be directed to the corresponding author.

## Author Contributions

ZG prepared **Figures 1**–**4** and wrote the manuscript text. YL and CL prepared **Figures 2**, **3**. HW prepared **Figure 1**. JF supervised the study and drafted the manuscript. GY conceived and designed the study, obtained funding and drafted the manuscript. All authors contributed to the article and approved the submitted version.

## Funding

This work was supported by grants from National Natural Science Foundation of China (http://www.nsfc.gov.cn) [81871618] and Shandong Provincial Major Scientific and Technological Innovation Project (MSTIP) [2019JZZY011012]. The funders had no role in study design, data collection and analysis, decision to publish, or preparation of the manuscript.

## Conflict of Interest

The authors declare that the research was conducted in the absence of any commercial or financial relationships that could be construed as a potential conflict of interest.
