# Supplementary material for: Staphylococcus aureus β-Hemolysin Up-Regulates the Expression of IFN-γ by Human CD56bright NK Cells
Source: Front Cell Infect Microbiol. 2021 Mar 29;11:658141. doi: 10.3389/fcimb.2021.658141 (PMC8039520; doi:10.3389/fcimb.2021.658141)
Supplement: Supplementary file 1 [file DataSheet_1.pdf]

Supplementary Material

Supplementary Figures

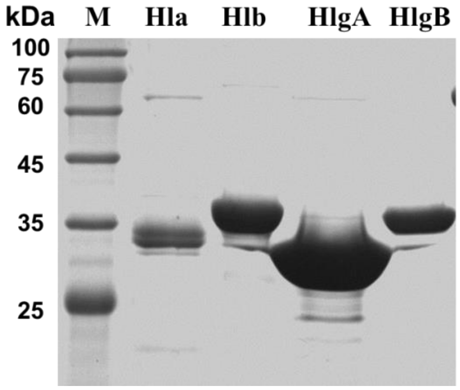

Supplementary Figure 1. The purified *S. aureus* hemolysins (Hla, Hlb and HlgA/HlgB) were determined by SDS-PAGE.

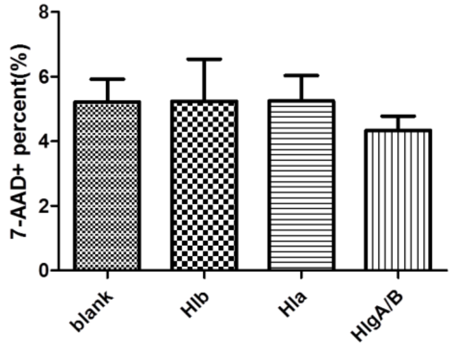

Supplementary Figure 2. Detection of lymphocytes death after incubation with different toxins. Human PBMC were incubated with Hlb (4μg/ml) or HlgA/B (4μg/ml) or Hla (0.25μg/ml) for 12 hours, respectively. Then cells were collected and stained with 7-AAD for flow cytometry analysis.

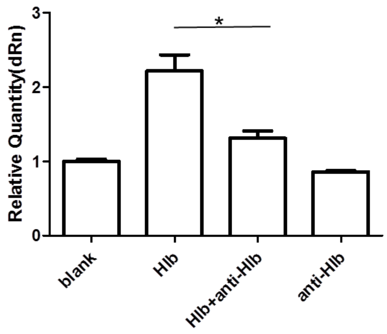

Supplementary Figure 3. IFN-γ mRNA expression assays. Anti-Hlb was pre-incubated with Hlb for 30mins at 37℃, and then NK-92 cells were incubated with different proteins for 12h, and IFN-γ mRNA expression was assayed by qRT-PCR. * means P<0.05.

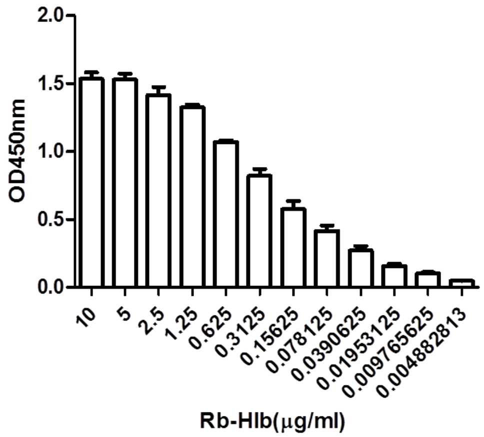

Supplementary Figure 4. The binding between Hlb and anti-Hlb was detected by ELISA.

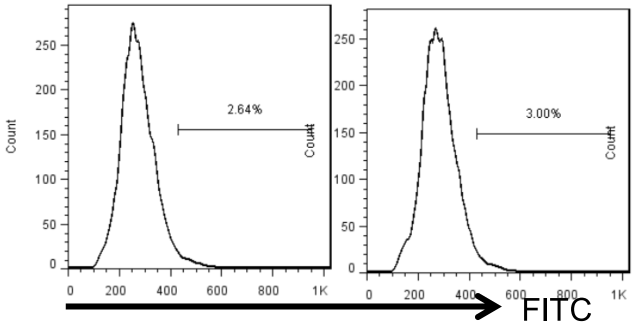

Supplementary Figure 5. The binding between Hlb to NK-92 cells. NK-92 cells were incubated with 10μg/ml Hlb for one hour. Then cells were collected and stained with anti-6xHis tag antibody (FITC) for 30 minutes. Finally, cells were washed and analyzed by flow cytometry.

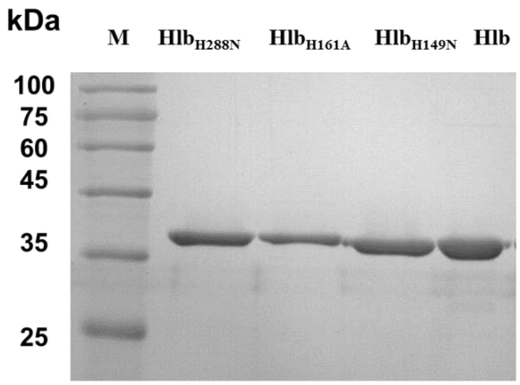

Supplementary Figure 6. The purified three Hlb mutants (Hlb$_{H288N}$, Hlb$_{H149N}$ and Hlb$_{H161A}$) were determined by SDS-PAGE.

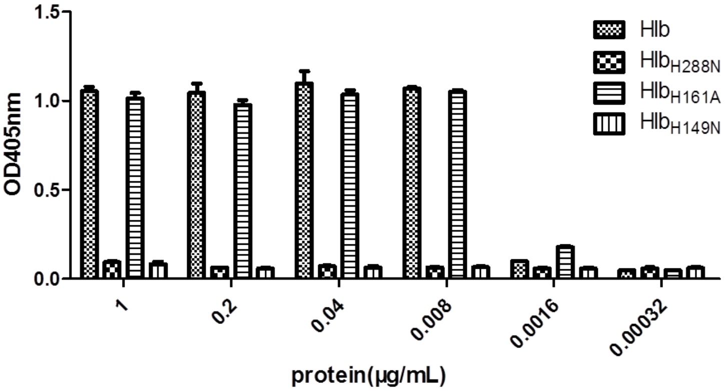

Supplementary Figure 7. Hemolytic activity assays. Sheep RBCs were incubated with four proteins (Hlb, Hlb$_{H288N}$, Hlb$_{H149N}$ and Hlb$_{H161A}$), and the released hemoglobin in the supernatants was detected at 405nm.

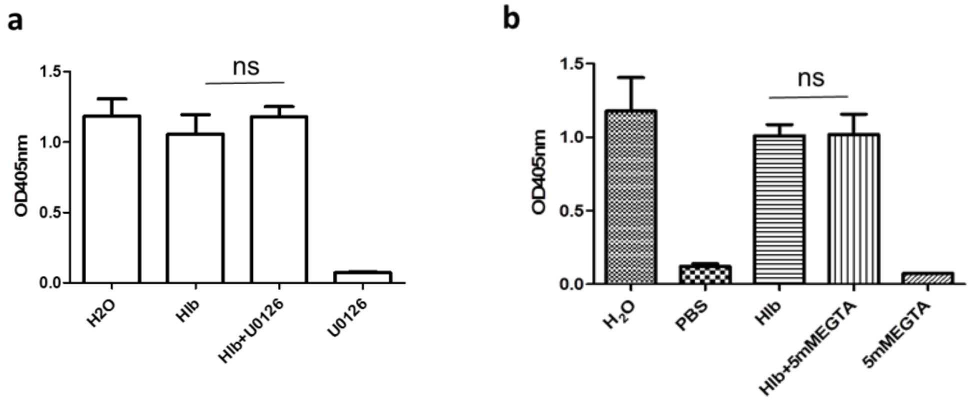

Supplementary Figure 8. (a, b) Hemolytic activity assays. Sheep RBCs were incubated with Hlb (2μg/ml) in the presence of 10μM U0126 (a) or 5mM EGTA (b). The released hemoglobin was detected at 405nm. ns, not significant.
